# Feature Reviews for Tomography 2023

**DOI:** 10.3390/tomography10100118

**Published:** 2024-10-09

**Authors:** Yashbir Singh, Emilio Quaia

**Affiliations:** 1 Department of Radiology, Mayo Clinic, Rochester, MN 55905, USA; 2 Department of Radiology, University of Padova, 35122 Padova, Italy

In an era of rapid technological progress, this Special Issue aims to provide a comprehensive overview of the state-of-the-art in tomographic imaging. We have curated a selection of papers that explore innovative applications of artificial intelligence in medical imaging, address critical concerns regarding patient and professional dose exposure, delve into the latest COVID-19 imaging research, and highlight breakthroughs in interventional radiology. By assembling this diverse range of topics, we seek to foster interdisciplinary dialog and inspire future research directions in the field of tomography. The insights presented in these reviews will not only serve as a valuable resource for researchers and clinicians but also pave the way for novel approaches to diagnosis, treatment planning, and patient care.

This collection brings together high-quality review papers from our esteemed Editorial Board Members and leading experts in the field, showcasing the latest advancements and cutting-edge developments in imaging science research.

This Special Issue of Tomography showcases a rich tapestry of cutting-edge research and expert reviews across various domains of imaging science: Several papers explore the transformative potential of AI in medical imaging, from enhancing image reconstruction algorithms to improving diagnostic accuracy in complex cases [1,2]. These studies demonstrate how machine learning and deep learning techniques are revolutionizing image analysis and interpretation [1,2]. A series of reviews addresses the critical issue of radiation exposure in medical imaging, presenting novel approaches to minimize patient and professional dose while maintaining image quality. These papers offer valuable insights into balancing diagnostic efficacy with safety considerations [3]. In light of the ongoing global health crisis, multiple studies focus on the latest developments in COVID-19 imaging. These reviews cover innovative techniques for early detection, disease progression monitoring, and long-term follow-up using various imaging modalities [4]. The Special Issue highlights recent advancements in interventional radiology, including novel image-guided procedures, real-time imaging techniques, and minimally invasive therapeutic approaches. These papers underscore the growing importance of interventional techniques in modern healthcare [5]. Several reviews explore cutting-edge developments in tomographic imaging, such as photon-counting CT, advanced MRI sequences, and hybrid imaging modalities. These papers provide insight into the future direction of imaging technologies and their potential clinical applications [2,6,7]. By covering these diverse topics, the Special Issue offers a comprehensive overview of the current state and future prospects of tomographic imaging science, serving as an invaluable resource for researchers, clinicians, and technologists in the field.

This Special Issue of Tomography highlights several key themes that are shaping the future of imaging science:

1. The increasing integration of artificial intelligence in medical imaging, enhancing both image quality and diagnostic capabilities.
2. A growing focus on radiation dose optimization, balancing diagnostic efficacy with patient and professional safety.
3. Rapid advancements in COVID-19 imaging techniques, contributing to improved diagnosis and management of the disease.
4. Continued innovation in interventional radiology, expanding the scope of minimally invasive, image-guided procedures.
5. Emergence of novel tomographic technologies that promise to revolutionize medical imaging and patient care.

These developments underscore the dynamic nature of the field and its potential to significantly impact healthcare delivery. The diverse range of topics covered in this Special Issue reflects the multidisciplinary nature of modern imaging science and the importance of collaborative research in driving innovation.

As we conclude this Special Issue, we invite researchers, clinicians, and imaging specialists to build upon the insights presented here and contribute to the ongoing advancement of tomographic imaging science. The field is ripe with opportunities for groundbreaking research and innovative applications.

We encourage readers to:

1. Explore collaborative opportunities that bridge the gap between technological innovation and clinical practice.
2. Pursue further research into the integration of artificial intelligence in medical imaging, particularly in areas that can enhance diagnostic accuracy and efficiency.
3. Investigate novel approaches to dose optimization that maintain or improve image quality while prioritizing patient and professional safety.
4. Continue developing and refining imaging techniques for COVID-19 and other emerging health challenges.
5. Push the boundaries of interventional radiology, exploring new applications and improving existing techniques.

Furthermore, we invite submissions for future issues of Tomography that build upon the themes explored in this Special Issue. Your contributions are vital in shaping the future of imaging science and improving patient care. Let us collectively strive to translate these scientific advancements into tangible improvements in healthcare delivery, ultimately benefiting patients worldwide. The future of tomographic imaging is bright, and your active participation will help illuminate the path forward.
